# How does pressure configuration affect Cobb angle during AIS brace casting?

**DOI:** 10.1186/1748-7161-10-S1-O60

**Published:** 2015-01-19

**Authors:** Eric Chalmers, Doug Hill, Andreas Donauer, Melissa Tilburn, Vicky Zhao, Edmond Lou

**Affiliations:** 1 University of Alberta, Edmonton, AB T6G 2R3, Canada; 2 Alberta Health Services, Canada

## Objectives

This work measured the magnitude and distance between pressures applied to the torso of AIS patients by an orthotist during brace casting. The instantaneous effect of the pressure configuration on Cobb angle was investigated using ultrasound. The objective was to use real-time measurements to confirm that pressure configuration affects Cobb angle predictably.

## Methods

Nine AIS patients undergoing casting for new braces participated in this pilot study (2 males, 7 females, aged 11-16, Cobb angles 16-44 degrees). An ultrasound scan was used to measure the patient's (baseline) Cobb angle. The orthotist then used a custom standing Providence system to apply corrective pressures - simulating a brace. A second ultrasound scan measured the new (corrected) Cobb angle. The orthotist could then try to achieve additional correction by adjusting the pressure magnitude/location. The process of adjusting pressures and ultrasound scanning repeated two or three times; the orthotist then chose the most satisfactory pressure configuration and performed the actual casting.

The procedure produced 26 individual scans (including baseline scans) from the 9 patients. The magnitude of applied pressures was measured using inflatable air bladders fixed to the Providence pads. The air pressure was measured during the ultrasound scan. The distance between pads was measured and multiplied by the total pressure to create a torque-like measurement.

Robust linear regression was used to relate pressure with Cobb angle correction, and torque with correction. Outlier points were removed if they fell more than 1.5 standard deviations from the regression line. Correlations between pressure/torque and correction were then measured.

## Results

Two outlier points were removed - both belonging to a single patient. Pressures ranged from 16-113 mmHg. The major curves' correction ranged from 0-39%. Significant correlations existed between average pressure and Cobb angle correction (r = 0.86, p < 0.01), and average torque and Cobb angle correction (r = 0.82, p < 0.01).

## Conclusions

Cobb angle correction can be increased by increasing the applied pressure. Curves which allow larger spacing between the pressure pads should also expect larger correction. This pilot study is the first to validate these concepts in real-time using noninvasive ultrasound technology.

